# Rapid detection of genetic mutations in individual breast cancer patients by next-generation DNA sequencing

**DOI:** 10.1186/s40246-015-0024-4

**Published:** 2015-02-08

**Authors:** Suqin Liu, Hongjiang Wang, Lizhi Zhang, Chuanning Tang, Lindsey Jones, Hua Ye, Liying Ban, Aman Wang, Zhiyuan Liu, Feng Lou, Dandan Zhang, Hong Sun, Haichao Dong, Guangchun Zhang, Zhishou Dong, Baishuai Guo, He Yan, Chaowei Yan, Lu Wang, Ziyi Su, Yangyang Li, Xue F Huang, Si-Yi Chen, Tao Zhou

**Affiliations:** The First Affiliated Hospital of Dalian Medical University, Dalian, Liaoning China; San Valley Biotechnology Incorporated, Beijing, China; Norris Comprehensive Cancer Center, Department of Molecular Microbiology and Immunology, Keck School of Medicine, University of Southern California, Los Angeles, CA USA

**Keywords:** Breast cancer, Genetic mutations, Ion torrent sequencing, Targeted therapy, Personalized medicine

## Abstract

Breast cancer is the most common malignancy in women and the leading cause of cancer deaths in women worldwide. Breast cancers are heterogenous and exist in many different subtypes (luminal A, luminal B, triple negative, and human epidermal growth factor receptor 2 (HER2) overexpressing), and each subtype displays distinct characteristics, responses to treatment, and patient outcomes. In addition to varying immunohistochemical properties, each subtype contains a distinct gene mutation profile which has yet to be fully defined. Patient treatment is currently guided by hormone receptor status and HER2 expression, but accumulating evidence suggests that genetic mutations also influence drug responses and patient survival. Thus, identifying the unique gene mutation pattern in each breast cancer subtype will further improve personalized treatment and outcomes for breast cancer patients. In this study, we used the Ion Personal Genome Machine (PGM) and Ion Torrent AmpliSeq Cancer Panel to sequence 737 mutational hotspot regions from 45 cancer-related genes to identify genetic mutations in 80 breast cancer samples of various subtypes from Chinese patients. Analysis revealed frequent missense and combination mutations in *PIK3CA* and *TP53*, infrequent mutations in *PTEN*, and uncommon combination mutations in luminal-type cancers in other genes including *BRAF*, *GNAS*, *IDH1*, and *KRAS*. This study demonstrates the feasibility of using Ion Torrent sequencing technology to reliably detect gene mutations in a clinical setting in order to guide personalized drug treatments or combination therapies to ultimately target individual, breast cancer-specific mutations.

**Electronic supplementary material:**

The online version of this article (doi:10.1186/s40246-015-0024-4) contains supplementary material, which is available to authorized users.

## Introduction

Breast cancer is the second most common malignancy worldwide and the most frequent in women. Roughly 1.67 million new cases and 522,000 deaths were reported globally in 2012, making breast cancer the fifth leading cause of cancer death. Breast cancer incidence differs with population and geographic location, where China alone accounted for more than 187,000 cases and nearly 48,000 deaths in 2012, whereas over 230,000 cases and more than 43,000 deaths were reported in the US [1]. Patient screening is superior in the US than in China [2], which may account for a higher incidence despite a much smaller population. While risk factors for developing breast cancer include ethnicity, older age, and environmental factors, lifestyle and diet also play a significant role, where westernization in Asia is thought to have contributed to the rise in spontaneous breast cancer incidence in Chinese populations over the last 20 years [3-5].

Breast cancers are highly heterogenous and may display different characteristics of hormone receptors (HR) (estrogen receptor (ER) and progesterone receptor (PR)) and human epidermal growth factor receptor 2 (HER2) status, and together this information helps to distinguish different types of breast cancers: luminal A (HR+/HER2−, tumor grade 1 or 2), luminal B/HER2− (HR+/HER2−, tumor grade 3 or 4), luminal B/HER2+ (HR+/HER2+), triple negative (HR−/HER2−), or HER2 overexpressing (HR−/HER2+) [6]. Together luminal A and B subtypes account for 65%–70% of all breast cancers, whereas 10%–15% are triple negative and 10%–20% are HER2 overexpressing [7].

These distinct types of breast cancers all have different characteristics, behaviors, and prognoses and also respond differently to drug treatments. Nearly three quarters of all breast cancers are ER+ and are therefore in some way dependent on estrogen for growth, providing a useful target for treating these cancers via ER modulators or downregulators or aromatase inhibitors [8]. But only 20%–40% of patients with advanced ER+ breast cancer have a response to endocrine therapy, which only averages 8 to 14 months [9]. Luminal A types tend to have the best outcome with a 95% 5-year survival rate, whereas luminal B tumors, which tend to have lower HR expression and subsequently less sensitivity to endocrine therapy but increased sensitive to chemotherapy, tend to have a worse outcome [10-12]. Typically found in younger patients, triple-negative breast cancers (TNBCs) are known to be particularly aggressive and are associated with germline *BRCA* mutations. TNBCs also have higher relapse rates and decreased overall patient survival than other breast cancer types [13]. HER2-overexpressing breast cancers also have poor prognoses and high metastases rates, and as they lack HR expression, they do not respond to endocrine therapies and are resistant to current chemotherapies [14]. These distinct breast cancer types can be further divided into a multitude of subtypes, and all of these types and subtypes exhibit distinct gene mutation patterns that have yet to be fully defined [10].

Currently, patient prognoses and treatment regimens for breast cancer are guided by the aforementioned characteristics of the tumor, but accumulating evidence suggests that this information is not enough; risk assessments, treatments, and patient outcomes are also influenced by both germline and somatic gene mutations. Known genetic factors like inherited *BRCA* mutations confer a lifetime risk of developing breast cancer of 60% to 85%; however, these mutations account for only 2%–3% of all breast cancer cases [15]. Spontaneous mutations in *PIK3CA* are a much more common event in breast cancers, with more than a quarter of breast cancer patients harboring a mutation in this gene [16,17]. While *PIK3CA* mutations have been shown to be associated with improved patient prognoses, these genetic aberrations have also been shown to impart resistance to trastuzumab, a common treatment option for HER2-overexpressing breast cancers [18]. Identifying gene mutations in patients with TNBC is especially important because these cancers currently do not have direct targets for treatments. Therefore, it is important to establish both the immunohistochemical properties and genetic profile of each breast cancer tumor to optimize treatment regimens and avoid unnecessary drug toxicities and ultimately to improve patient outcomes.

There are a number of different next-generation sequencing (NGS) platforms available, including Illumina, 454, and SOLiD, but these are typically expensive both in instrument and assay cost, and therefore, these tools are unrealistic for widespread clinical diagnostic use. But new technology like the Ion Torrent sequencing platform has been shown to be more cost and time effective with reliable results [19], which may help make cancer DNA sequencing and personalized treatments a reality for each cancer patient in the near future. In the present study, we have used Ion Torrent sequencing technology with the Ion Personal Genome Machine (PGM) and Ion Torrent AmpliSeq Cancer Panel as a rapid and affordable method to detect gene mutations in 80 clinical breast cancer samples of different types from Chinese patients.

## Materials and methods

### Ethics statement

The study has been approved by the Human Research Ethics Committee of the First Affiliated Hospital of Dalian Medical University, China. The institutional ethics committee waived the need for consent for formalin-fixed, paraffin-embedded (FFPE) tumor samples obtained from the tumor tissue bank at the hospital’s Department of Pathology. All samples and medical data used in this study have been irreversibly anonymized.

### Patient information

Tumor samples used in the study were collected from the First Affiliated Hospital of Dalian Medical University, China. A total of 80 FFPE tumor samples from Chinese breast cancer patients were analyzed (Table 1). Patients were an average of 55 years old, with a range of 30–75 years. American Joint Committee on Cancer (AJCC)/tumor, node, and metastasis (TNM) cancer staging was assessed, and tumor samples were also analyzed for immunohistochemical status of HR and HER2 (Table 1). Based on these, patients were categorized into five breast cancer subtypes: luminal A (HR+/HER2−, AJCC stage 1 or 2; 26.3%), luminal B/HER2− (HR+/HER2−, AJCC stage 3 or 4; 21.3%), luminal B/HER2+ (HR+/HER2+; 30.0%), triple negative (HR−/HER2−; 7.5%), and HER2 overexpressing (HR−/HER2+; 8.8%), and five samples (6.3%) were unclassifiable due to unknown HER2 status (Table 2).

**Table 1:** **Clinical features of 80 breast cancer patients**

**Characteristic**		***n*** **(%)**
Age (years)	Median: 56	
	Range: 30–75	
HR status	HR+	65 (81.3%)
	HR−	15 (18.8%)
HER2 status	HER2+	12 (15.0%)
	HER2++	18 (22.5%)
	HER2+++	1 (1.3%)
	HER2−	44 (55.0%)
	Unknown	5 (6.3%)
AJCC/TNM stage	2a	19 (23.8%)
	2b	26 (32.5%)
	3a	23 (28.8%)
	3b	4 (8.8%)
	3c	7 (8.8%)
	4	1 (1.3%)
Pathological diagnosis of infiltrating ductal carcinoma	IDC2	71 (88.8%)
	IDC3	6 (7.5%)
	Others	3 (3.8%)

**Table 2:** **Average patient age, average disease-free survival (DFS), and mutation frequency in breast cancer subtypes with or without mutations**

**Subtype**	***n*** **(% total)**	**Average age (years)**	**Average DFS (months)**	**Samples with mutations (freq.)**	**Samples without mutations (freq.)**	**Average age with mutations (years)**	**Average age without mutations (months)**	***P*** **value**	**Average DFS with mutations (months)**	**Average DFS without mutations (months)**	***P*** **value**
All	80 (100%)	54.5	38.3	32 (40.0%)	48 (60.0%)	54.9	54.2	0.757	38.8	38.0	0.741
Luminal A	21 (26.3%)	53.7	39.0	9 (42.9%)	12 (57.1%)	53.8	53.7	0.979	40.6	37.8	0.407
Luminal B/HER2−	17 (21.3%)	55.8	39.4	6 (35.3%)	11 (64.7%)	57.3	54.9	0.626	40.5	38.7	0.799
Luminal B/HER2+	24 (30.0%)	52.9	37.3	11 (45.8%)	13 (54.2%)	53.6	52.3	0.772	37.5	37.1	0.936
Triple negative	6 (7.5%)	53.8	37.5	4 (66.7%)	2 (33.3%)	58.8	44.0	0.165	39.8	33.0	0.508
HER2 overexpressing	7 (8.8%)	56.6	34.6	2 (28.6%)	5 (71.4%)	52.5	58.2	0.633	31.5	35.8	0.595
Unknown	5 (6.3%)	59.0	43.0	0 (0.0%)	5 (100%)	-	59.0	-	-	43.0	-

### DNA preparation

FFPE tissue samples were first deparaffinized in xylene, 3–5-μm-thick sections were extracted, and DNA was isolated using the QIAamp DNA Mini Kit (QIAGEN) as per the manufacturer’s instructions.

### Ion Torrent PGM library preparation and DNA sequencing

An Ion Torrent adapter-ligated library was constructed with the Ion AmpliSeq Library Kit 2.0 (Life Technologies, Part #4475345 Rev. A) following the manufacturer’s protocol. Briefly, 50 ng of pooled amplicons were end-repaired, Ion Torrent adapters P1 and A were ligated, and the adapter-ligated products were then purified with AMPure beads (Beckman Coulter, Brea, CA, USA), nick-translated, and PCR-amplified for 5 cycles. The resulting library was purified with AMPure beads (Beckman Coulter), and the library concentration and size was determined with the Agilent 2100 Bioanalyzer and Agilent Bioanalyzer DNA High-Sensitivity LabChip (Agilent Technologies).

Sample emulsion PCR, emulsion breaking, and enrichment were performed with the Ion PGM 200 Xpress Template Kit (Life Technologies, Part #4474280 Rev. B), according to the manufacturer’s instructions. Briefly, an input concentration of one DNA template copy/Ion Sphere Particles (ISPs) was added to emulsion PCR master mix, and the emulsion was generated with an IKADT-20 mixer (Life Technologies). Next, ISPs were recovered, and template-positive ISPs were enriched with Dynabeads MyOne Streptavidin C1 beads (Life Technologies). The Qubit 2.0 fluorometer (Life Technologies) was used to confirm ISP enrichment. Three-hundred sixteen chips were used to sequence barcoded samples on the Ion Torrent PGM for 65 cycles, and an Ion PGM 200 Sequencing Kit (Life Technologies, Part # 4474004 Rev. B) was used for sequencing reactions, as per the recommended protocol.

This Personalized Cancer Mutation Panel targets 737 mutational hotspot regions in the following 45 genes: *ABL1*, *AKT1*, *ALK*, *APC*, *ATM*, *BRAF*, *CDH1*, *CDKN2A*, *CSF1R*, *CTNNB1*, *EGFR*, *ERBB2*, *ERBB4*, *FBXW7*, *FGFR1*, *FGFR2*, *FGFR3*, *FLT3*, *GNAS*, *HNF1A*, *HRAS*, *IDH1*, *JAK3*, *KDR*, *KIT*, *KRAS*, *MET*, *MLH1*, *MPL*, *NOTCH1*, *NPM1*, *NRAS*, *PDGFRA*, *PIK3CA*, *PTEN*, *PTPN11*, *RB1*, *RET*, *SMAD4*, *SMARCB1*, *SMO*, *SRC*, *STK11*, *TP53*, and *VHL*.

### Variant calling

Initial data from the PGM runs were processed with the Ion Torrent platform-specific pipeline software Torrent Suite to generate sequence reads, then trim adapter sequences, filter, and remove poor signal-profile reads. Torrent Suite software v3.4 with a plug-in “variant caller v3.4” program was used to generate initial variant calling from the Ion AmpliSeq sequencing data. In order to eliminate erroneous base calling and generate final variant calling, several filtering steps were used: defining average total coverage depth, variant coverage, variant frequency of each sample, and *P* value <0.01; visually inspecting and removing DNA strand-specific errors; defining variants within hotspots; and eliminating variants in amplicon AMPL339432 (PIK3CA, exon13, chr3:178938822–178938906) which is not uniquely matched in the human genome.

### Sequence coverage

**Figure 1:**
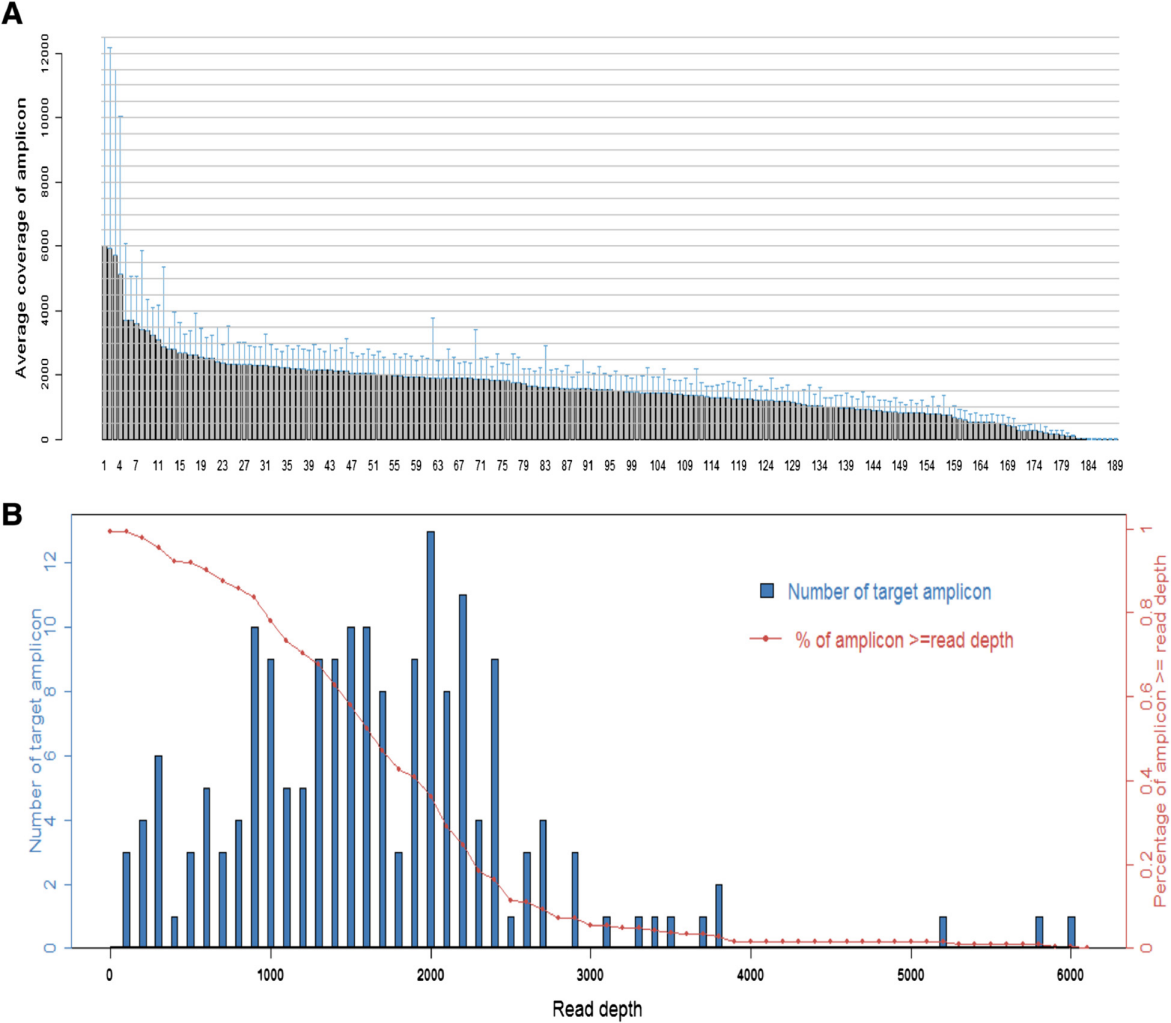
**Sequence read distribution across 189 amplicons generated from 80 breast cancer samples, normalized to 300,000 reads per sample. (A)** Average number of reads observed for each amplicon. **(B)** Number of targets with a given read depth, sorted in bins of 100 reads.

From the 80 samples, the mean read length was 76 bp and the average reads were approximately 24 Mb of sequence per sample. With normalization to 300,000 reads per specimen, there was an average of 1,639 reads per amplicon (range: 22 to 6,020) (Figure 1A); 180/189 (95.2%) amplicons averaged at least 100 reads; and 170/189 (89.9%) amplicons averaged at least 300 reads (Figure 1B).

### Somatic mutations

Detected mutations were compared to variants in the 1000 Genomes Project [20] and 6,500 exomes of the National Heart, Lung, and Blood Institute Exome Sequencing Project [21] to distinguish between somatic and germline mutations.

### Bioinformatical and experimental validation

We used the COSMIC [22] (version 64), MyCancerGenome database (http://www.mycancergenome.org/), and some publications to assess recurrent mutations in breast cancer (Additional file 1: Table S1). Additionally, detected missense mutations were confirmed by Sanger sequencing (data not shown). All mutations identified with Sanger sequencing were consistent with those identified with the Ion Torrent PGM.

### Statistical analysis

The Fisher’s exact test was used to define significant values in the detected mutated genes, and the total variants and odds ratios (OR) between samples with mutations and without mutations were determined using 2 × 2 contingency tables and the GraphPad QuickCalcs online calculator for Scientists (http://www.graphpad.com/quickcalcs/index.cfm). All *P* values are two-sided, and statistical significance was defined as *P* < 0.05.

## Results and discussion

**Figure 2:**
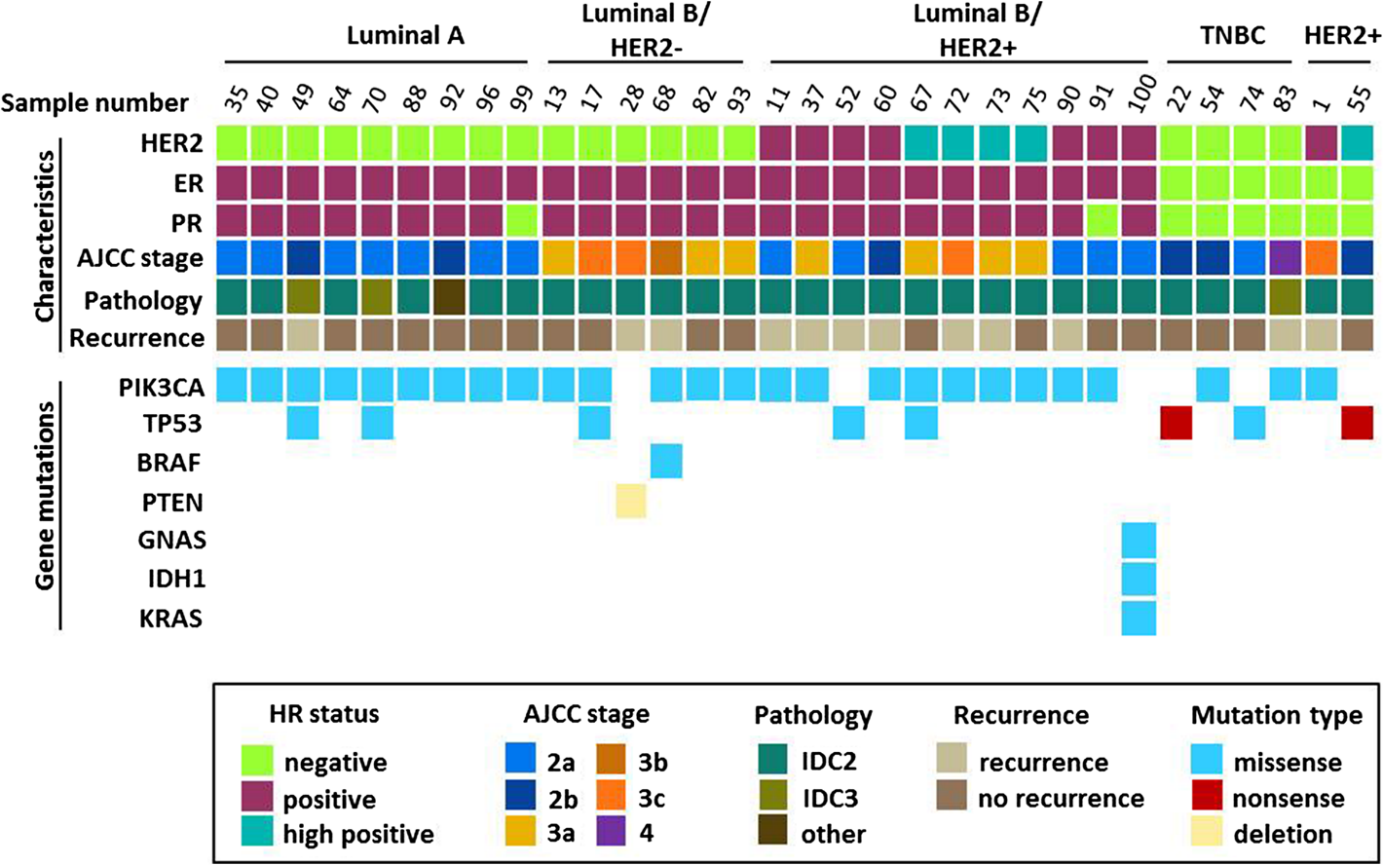
**Summary of mutated genes detected in 80 breast cancer samples.** Thirty-two samples harbor mutations in *PIK3CA*, *TP53*, *KRAS*, *BRAF*, *PTEN*, *GNAS*, and *IDH1*. Samples are classified by four methods: 1) Immunohistochemistry of ER, PR, and HER2; 2) pathologic type (IDC2, IDC3, other); 3) AJCC/TNM-staging (2a, 2b, 3a, 3b, 3c, 4); and 4) recurrence or no recurrence.

**Figure 3:**
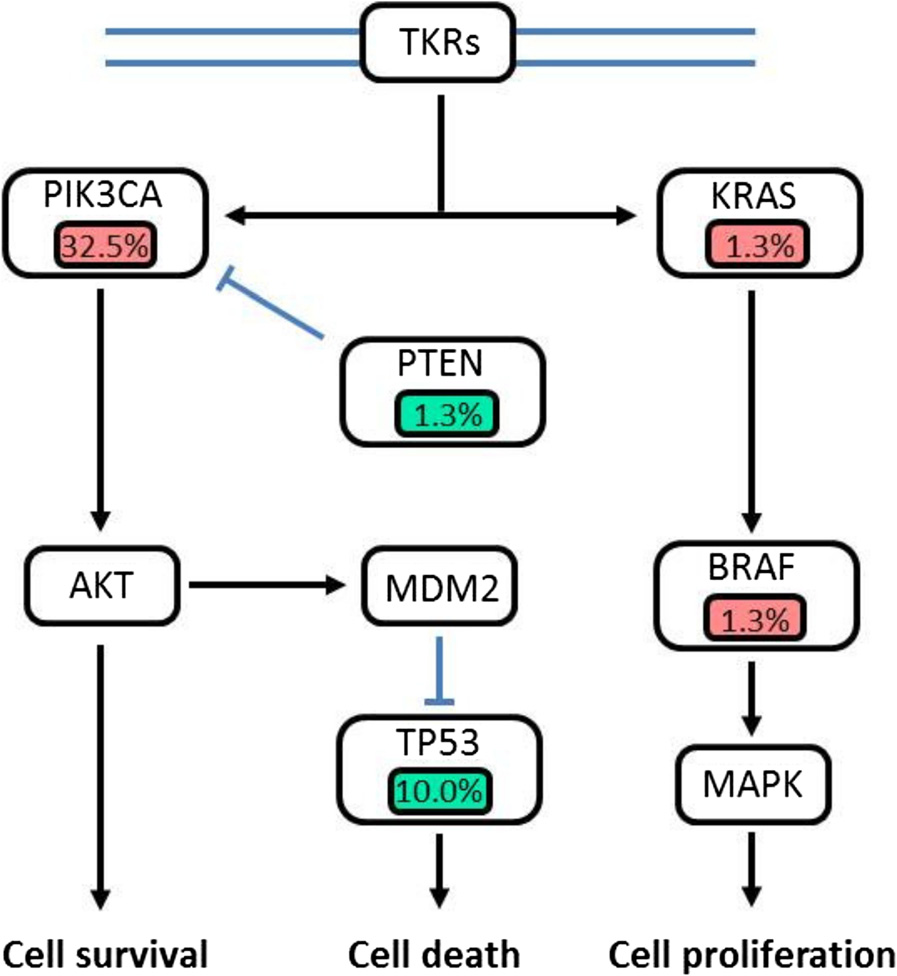
**Signaling pathways in breast cancer with gene mutations.** Genetic alterations in breast cancer primarily occur in genes of the MAPK, p53, and PI3K/AKT signaling pathways. Alterations in oncogenes are indicated in pink, and those in cancer suppressor genes are shown in green. Percentages (%) are the frequency of mutations per gene in our study out of 80 samples. Tyrosine kinase receptors (TKRs) include HER2, EGFR, and IGF-1R in breast cancer.

From the 45 genes screened in our study, 39 mutations were detected in 32 of 80 samples (40.0%) (Figure 2). Except for the five unclassified samples with no mutations, mutations were detected at different frequencies across all breast cancer subtypes (Table 2). Triple negative samples contained the highest mutation frequency (66.7%), whereas HER2-overexpressing samples contained the lowest mutation frequency (28.6%), and luminal A, luminal B/HER2−, and luminal B/HER2+ had similar mutation frequencies (42.9%, 35.3%, and 45.8%, respectively). Twenty-six samples (32.5%) contained one mutation, five samples (6.3%) contained two mutations, and one sample (1.3%) contained three mutations, and interestingly, combination mutations were only found in the luminal subtypes (Table 3). *PIK3CA* mutations and TP53 mutations were the most prevalent (32.5% and 10.0%, respectively), and mutations were also identified in *BRAF*, *GNAS*, *IDH1*, *KRAS*, and *PTEN* all at a frequency of 1.3% (Figure 3). Among each subtype, there was no statistically significant difference in age or disease-free survival (DFS) between patients with mutations and patients without mutations (Table 2).

**Table 3:** **Detected point mutations per breast cancer subtype**

**Breast cancer type**	**Gene(s)**	**Mutation(s)**	**Age (years)**	**DFS (months)**	**Recurrence**	**AJCC**	**ER**	**PR**	**HER2**
Luminal A	*PIK3CA*	p.N345K	62	36	N	2a	+	+	−
	*PIK3CA*	p.E545K	46	36	N	2a	+	+	−
	*PIK3CA*	p.E545K	61	47	N	2a	+	+	−
	*PIK3CA*	p.H1047L	55	36	N	2b	+	+	−
	*PIK3CA*	p.H1047R	51	48	N	2a	+	+	−
	*PIK3CA*	p.H1047R	46	42	N	2a	+	+	−
	*PIK3CA*	p.H1047R	56	36	N	2a	+	−	−
	*PIK3CA/TP53*	p.H1047R/p.R248W	57	41	N	2a	+	+	−
	*PIK3CA/TP53*	p.H1047R/p.R175H	50	36	Y	2b	+	+	−
Luminal B/HER2−	*PTEN*	p.T321fs*23	55	28	Y	3c	+	+	−
	*PIK3CA/TP53*	p.E545K/ p.H193R	52	53	N	3c	+	+	−
	*PIK3CA/BRAF*	p.H1047R/ p.V600M	59	36	Y	3b	+	+	−
	*PIK3CA*	p.H1047R	52	36	N	3a	+	+	−
	*PIK3CA*	p.H1047R	58	36	N	3a	+	+	−
	*PIK3CA*	p.H1047R	68	54	N	3a	+	+	−
Luminal B/HER2+	*GNAS/IDH1/KRAS*	p.R201C/ p.R132C/ p.G12D	57	36	N	2a	+	+	+
	*PIK3CA*	p.E542K	50	47	N	3a	+	+	+
	*PIK3CA*	p.E545K	66	42	N	2b	+	+	+
	*PIK3CA*	p.H1047L	72	40	N	3a	+	+	++
	*PIK3CA*	p.H1047R	44	36	N	2a	+	+	+
	*PIK3CA*	p.H1047R	45	55	N	2a	+	+	+
	*PIK3CA*	p.H1047R	32	36	N	2a	+	−	+
	*PIK3CA*	p.H1047R	65	25	Y	3a	+	+	++
	*PIK3CA*	p.H1047R	63	12	Y	3c	+	+	++
	*PIK3CA/TP53*	p.H1047R/ p.P278L	49	41	N	3a	+	+	++
	*TP53*	p.Y220C	47	43	N	2a	+	+	+
Triple negative	*PIK3CA*	p.E545K	60	43	N	2b	−	−	−
	*PIK3CA*	p.E542K	61	26	Y	4	−	−	−
	*TP53*	p.Y163C	57	40	N	2a	−	−	−
	*TP53*	p.R196*	57	50	N	2b	−	−	−
HER2 overexpressing	*PIK3CA*	p.H1047R	65	20	Y	3c	−	−	+
	*TP53*	p.R213*	40	43	N	2b	−	−	++

*DFS* disease-free survival, *del* deletion, *fs* frameshift.

*Nonsense mutation resulting in a stop codon.

### *PIK3CA* mutations

Twenty-six samples (32.5%) harbored *PIK3CA* mutations, which accounted for 66.7% of all detected mutations in our study. Five different *PIK3CA* mutations were identified: p.N345K in the C2 domain encoded by exon 4, p.E542K and p.E545K in the helical domain encoded by exon 9, and p.H1047R and p.H1047L in the kinase domain encoded by exon 20. Mutations p.E542K, p.E545K, and p.H1047R have been found in previous studies to be the most prevalent in human breast cancers and are associated with an increase in kinase activity in the PI3K pathway [23,24]. These three mutations accounted for 88.5% of all *PIK3CA* mutations in our study. The remaining mutations, p.N345K and p.H1047L, are much less common and are found in less than 2% and 3.5% of breast cancers, respectively [22,25,26].

*PIK3CA* mutations are an early event in breast cancer development. Accordingly, we found mutations in this gene at all stages and all breast cancer subtypes in our study. While previous research has found *PIK3CA* mutations to be associated with older patient age [25], we did not find significant differences between age and *PIK3CA* mutations among all patients or breast cancer subtypes. Consistent with a study by Kalinsky et al. [25], *PIK3CA* mutations were found in more HER2− tumors than HER2+ tumors (61.5% vs. 38.5%, respectively), although not significantly (OR: 1.20; *P* = 0.81). Also, patients with HR+ tumors had a higher likelihood of having a PIK3CA mutation than those with HR− tumors, but again, this was not statistically significant (OR: 2.19; *P* = 0.36). Others have found lower *PIK3CA* mutation frequencies in luminal B than luminal A breast cancers [10], but we found the opposite in our study with 53.8% of PIK3CA-mutated samples as luminal B types vs. 34.6% luminal A type. Drugs like everolimus, a rapamycin derivative that inhibits PI3K/ATK/mTOR signaling, have been approved to treat advanced HR+/HER2− breast cancer patients after other treatments have failed and have been shown to increase progression-free survival [27-29].

While the study of Kalinsky et al. found overall patient survival to be significantly improved in patients with *PIK3CA* mutations [25], we found that patients with *PIK3CA* mutations collectively had a roughly equal DFS time to patients with WT *PIK3CA* (38.6 vs. 38.2 months, respectively); however, patients with triple negative and HER2-overexpressing tumors and *PIK3CA* mutations had a shorter average DFS (34.5 and 20 months, respectively) than those with WT *PIK3CA* (39 and 37 moths, respectively), possibly suggesting that the other characteristics of the tumor may have greater prognostic value than *PIK3CA* mutations. Consistent with a clinical study on the impact of specific *PIK3CA* mutations on breast cancer patient prognosis [30], we found that patients with *PIK3CA* mutations in exon 20 had a slightly shorter DFS than those with *PIK3CA* mutations in exons 4 and 9 (37.4 vs. 41.3 months, respectively; *P* = 0.37). Because activation of the PI3K/ATK/mTOR pathway has been shown to confer resistance to trastuzumab treatment in HER2+ breast tumors [31,32], and because the specific mutation may offer prognostic value, it is important to identify *PIK3CA* mutations in all breast cancer patients regardless of subtype.

### *TP53* mutations

Eight samples (10.0%) were found to harbor *TP53* mutations, and these mutations were identified in each subtype: 2 (9.5%) in luminal A, 1 (5.9%) in luminal B/HER2−, 2 (8.3%) in luminal B/HER2+, 2 (33.3%) in triple negative, and 1 (14.3%) in HER2-overexpressing tumors. There was no statistically significant difference between age and *TP53* mutations among all patients or breast cancer subtypes, nor was there a difference in DFS and *TP53* mutations and subtype. Among 80 samples, we found *TP53* mutations were more likely to occur in HR− tumors (OR: 3.0; *P* = 0.17) and tumors of a lower grade (75.0% at stage 2 vs. 25.0% at 3 or 4; OR: 2.54; *P* = 0.46). Five of the eight (62.5%) *TP53*-mutated tumors were HER2−, although study wide, there was no correlation between *TP53* mutations and HER2 status (OR: 1.25; *P* = 1.0). Others have reported that *TP53* is associated with worse overall patient survival [33-35], but we found this to be the opposite in our study; across all types, patients with *TP53* mutations had an average DFS of 44.3 vs. 37.7 months for those with WT *TP53* (*P* = 0.09).

The eight different *TP53* mutations detected in our study were found at known hotspot locations, all within the DNA binding domains: two in exon 5 (p.Y136C and p.R175H); four in exon 6 (p.H193R, p.R196*, p.R213*, and p.Y220C); one in exon 7 (p.R248W); and one in exon 8 (p.P278L). Mutations in exons 5 and 7 have previously been shown to correlate with poorer overall survival and disease-free progression in breast cancer patients [36,37]. Specifically, mutations in these exons which affect the L2 and L3 loop domains of the protein (codons 163–195 and 236–251, respectively) have been found to confer resistance to certain cytotoxic drugs, including 5-fluorouracil and mitomycin [38,39]. However, in our study, there were only four patients with *TP53* mutations in these exons and specific codons, and these patients had an equal DFS to patients with other *TP53* mutations (44.3 months), and a longer, albeit not significant, DFS than the study average of 38.3 months (*P* = 0.26).

### Combination and less frequent mutations

One advanced luminal B/HER2− sample contained a deletion in codon 321, exon 8 of *PTEN*, resulting in a frameshift mutation (p.T321fs*23). This mutation has been found in other cancers of the endometrium and large intestine but has not yet been identified in breast cancers [22]. As an antagonist of PI3K signaling, improper *PTEN* function leads to uncontrolled activation of its downstream signals [16], and reduced *PTEN* expression correlates with breast cancer progression and is associated with advanced disease [40,41]. Accordingly, the *PTEN*-mutated sample in our study was stage 3C, and the patient’s DFS was 10 months shorter that the study average. Others have reported that *PTEN* mutations occur more often in HR− tumors [41,42], which was not the case for the *PTEN*-mutated sample in our study.

Six of the samples (7.5%) contained mutations in a combination of genes, and all co-mutations were found in the luminal subtypes (100% HR+, 66.7% HER2−) (Table 3). Five of the six combination mutations co-occurred with *PIK3CA*, and four of these were a combination of *PIK3CA* and *TP53*. Research suggests that the presence of *PIK3CA* and *TP53* co-mutations increase breast cancer sensitivity to some PI3K/AKT/mTOR inhibitors when compared to those with *PIK3CA* mutations alone [43,44]; regardless, promising drugs like BEZ235, a PI3K/mTOR kinase inhibitor, may only be effective in a specific subset of triple-negative breast cancers [45].

One luminal B/HER2− sample harbored a combination mutation in *PIK3CA* (p.H1047R) and *BRAF* (p.V600M). This combination is commonly found in melanoma but rarely in breast cancer [46]. The vast majority of *BRAF* mutations identified in all cancer types are activating mutations that occur at codon 600, most commonly p.V600E (84.6%), whereas p.V600M accounts for only 0.3% of *BRAF* mutations at this codon [47]. While this mutation is not common in breast cancers, several drugs have been developed to target *BRAF* mutations in other cancer types and are showing promising results in breast cancer models [48,49]. By combining treatments to target both *PIK3CA* and *BRAF* mutations, patients with combination mutations such as these may find benefit greater than a single treatment. Everolimus, however, would not be effective because research has shown this drug to be ineffective in the presence of a *KRAS* or *BRAF* mutation [27].

One stage 2A luminal B/HER2+ sample contained a unique combination of mutations in three different genes, *GNAS* (p.R201C), *IDH1* (p.R132C), and *KRAS* (p.G12D), a combination that, to our knowledge, has yet to be identified in breast cancer. *GNAS* mutations, which are commonly found in pituitary and pancreatic cancers, and *IDH1* mutations, common in gliomas, are uncommon events in breast cancers, both found in less than 1% [22,50]. *KRAS* mutations are only slightly more common, found in 2%–5% of breast cancers [22,51]. *KRAS* is involved in the *EGFR* signaling pathway, and anti-EGFR drugs like lapatinib used to treat advanced HER2+ breast cancers require WT *KRAS* to be effective [52,53]. For a patient with these tumor characteristics, knowledge of mutated *KRAS* status would prevent unnecessary drug toxicity.

## Conclusion

Characterization of breast cancer tumors is critical in determining appropriate treatment options and predicting patient prognosis. The hormone receptor status and HER2 expression act as markers to direct drug treatments or to predict behavior of the disease. In addition to immunohistochemical properties, it is becoming increasingly evident that gene mutations play a role in breast cancer progression and response to treatment, making it critical to determine the genetic profile of each breast cancer tumor to personalize treatments and optimize patient outcomes. In our study, we used Ion Torrent sequencing technology to identify mutations in 80 clinical breast cancer tumors of various subtypes. Our study revealed not only uncommon and novel combination mutations but also mutations commonly found in breast cancers at frequencies similar to those previously reported, indicating the reliability of the Ion Torrent sequencing method to genotype cancer samples in a clinical setting. Ion Torrent sequencing technology has also been shown to be more cost and time effective than other traditional sequencing methods [19,54], and as such, it may be a feasible way to advance personalized patient treatments by providing clinicians a tool to characterize breast cancers beyond immunohistochemical markers and ultimately improve outcomes for breast cancer patients.

## Additional file

Additional file 1: Table S1. Frequencies of missense point mutations, insertion, and deletion mutations in 737 mutational hotspot regions of 45 genes in 80 breast cancer samples.
