# Supplementary material for: Rapid detection of genetic mutations in individual breast cancer patients by next-generation DNA sequencing
Source: Hum Genomics. 2015 Feb 8;9(1):2. doi: 10.1186/s40246-015-0024-4 (PMC4348109; doi:10.1186/s40246-015-0024-4)
Supplement: Additional file 1: Table S1. — Frequencies of missense point mutations, insertion, and deletion mutations in 737 mutational hotspot regions of 45 genes in 80 breast cancer samples. [file 40246_2015_24_MOESM1_ESM.docx]

**Additional file 1: Table S1.** Frequencies of missense point mutations, insertion and deletion mutations in 737 loci of 45 genes in 80 breast cancer samples.

| **Gene Mutations** | **Number of samples with this mutation site** | **Number of samples with this mutation gene** | **Mutation Frequency** | **Gene mutation frequency in some publications** | **Site mutation frequency in gene in some publications** | **If reported in breast cancer in COSMIC database** | **If reported in breast cancer in MyCancerGenome** |
| --- | --- | --- | --- | --- | --- | --- | --- |
| BRAF c.1798G>A | 1 | 1 | 1.3% | 2%[1] (England) |  | YES | NO |
| GNAS c.601C>T | 1 | 1 | 1.3% |  |  | NO | NO |
| IDH1 c.394C>T | 1 | 1 | 1.3% |  |  | NO | NO |
| PIK3CA c.1035T>A | 1 | 26 | 32.5% | 26.0% |  | YES | NO |
| PIK3CA c.1624G>A | 2 |  |  |  | up to 11% | YES | YES |
| PIK3CA c.1633G>A | 5 |  |  |  | up to 20% | YES | YES |
| PIK3CA c.3140A>G | 16 |  |  |  | up to 55% | YES | YES |
| PIK3CA c.3140A>T | 2 |  |  |  | up to 5% | YES | YES |
| KRAS c.35G>A | 1 | 1 | 1.3% | <1%[2] (Finnish) | <1%[2] | YES | NO |
| PTEN c.963delA | 1 | 1 | 1.3% | 7.0% |  | NO | NO |
| TP53 c.488A>G | 1 | 8 | 10.0% | 40%[3] (England) |  | YES | NO |
| TP53 c.524G>A | 1 |  |  |  |  | YES | NO |
| TP53 c.578A>G | 1 |  |  |  |  | YES | NO |
| TP53 c.586C>T | 1 |  |  |  |  | YES | NO |
| TP53 c.637C>T | 1 |  |  |  |  | YES | NO |
| TP53 c.659A>G | 1 |  |  |  |  | YES | NO |
| TP53 c.742C>T | 1 |  |  |  |  | YES | NO |
| TP53 c.833C>T | 1 |  |  |  |  | YES | NO |

REFERENCES

1. Davies H, Bignell GR, Cox C, Stephens P, Edkins S, et al. (2002) Mutations of the BRAF gene in human cancer. Nature 417: 949-954.

2. Loi S, Michiels S, Lambrechts D, Fumagalli D, Claes B, et al. (2013) Somatic mutation profiling and associations with prognosis and trastuzumab benefit in early breast cancer. J Natl Cancer Inst 105: 960-967.

3. Coles C, Condie A, Chetty U, Michael Steel C, John Evans H, et al. (1992) p53 Mutations in Breast Cancer. Cancer Research 52: 5291-5298.
